# Association Between Drug Treatments and the Incidence of Liver Injury in Hospitalized Patients With COVID-19

**DOI:** 10.3389/fphar.2022.799338

**Published:** 2022-03-21

**Authors:** Suyu Gao, Qingqing Yang, Xuanxuan Wang, Wen Hu, Yun Lu, Kun Yang, Qiaoli Jiang, Wenjing Li, Haibo Song, Feng Sun, Hong Cheng

**Affiliations:** ^1^ Department of Pharmacy, Zhongnan Hospital of Wuhan University, Wuhan, China; ^2^ Department of Epidemiology and Biostatistics, School of Public Health, Peking University, Beijing, China; ^3^ Key Laboratory for Research and Evaluation of Pharmacovigilance, National Medical Products Administration, Beijing, China; ^4^ Chinese Society of Toxicology, Beijing, China

**Keywords:** COVID-19, drug-induced liver injury (DILI), drug treatment, risk factors, multi-center retrospective study

## Abstract

The outbreak of coronavirus disease 2019 (COVID-19) has led to the emergence of global health care. In this study, we aimed to explore the association between drug treatments and the incidence of drug-induced liver injury (DILI) in hospitalized patients with COVID-19. A retrospective study was conducted on 5113 COVID-19 patients in Hubei province, among which 395 incurred liver injury. Hazard ratios (HRs) and 95% confidence intervals (CIs) were estimated by Cox proportional hazards models. The results showed that COVID-19 patients who received antibiotics (HR 1.97, 95% CI: 1.55–2.51, *p* < 0.001), antifungal agents (HR 3.10, 95% CI: 1.93–4.99, *p <* 0.001) and corticosteroids (HR 2.31, 95% CI: 1.80–2.96, *p* < 0.001) had a higher risk of DILI compared to non-users. Special attention was given to the use of parenteral nutrition (HR 1.82, 95% CI: 1.31–2.52, *p* < 0.001) and enteral nutrition (HR 2.71, 95% CI: 1.98–3.71, *p* < 0.001), which were the risk factors for liver injury. In conclusion, this study suggests that the development of DILI in hospitalized patients with COVID-19 needs to be closely monitored, and the above-mentioned drug treatments may contribute to the risk of DILI.

## Introduction

The outbreak of Coronavirus Disease 2019 (COVID-19) has become the greatest threat to human health worldwide, which is caused by the severe acute respiratory syndrome coronavirus 2 (SARS-CoV-2) (Huang et al., 2020). Clinical symptoms of COVID-19 range from mild symptoms of fever, cough, shortness of breath and fatigue to severe pneumonia requiring admission to an intensive care unit (ICU) and mechanical ventilation (Patel et al., 2020). Although several vaccines have been developed, due to uncertainty around the effectiveness of a vaccine and its global uptake, the development of novel therapeutic agents is still a challenge and in great demand.

Liver injury occurs in some patients with COVID-19, and its severity varies depending on patient age, geographical area, and disease severity (Huang et al., 2020). Liver is the primary organ for metabolism and detoxification; thus; maintaining healthy liver function is critical to engage in the treatment regimens for COVID-19 (Amin, 2021; Yu et al., 2021). Since most of the drugs are metabolized in the liver, liver injury still occurs even though they are primarily consumed for therapeutic purposes. Hospitalized patients with COVID-19 are often poly-medicated, which significantly increases their risks of drug-drug interactions and drug-induced adverse events (Barlow et al., 2020; Li and Fan, 2020). Various potentially hepatotoxic medications have been used in these patients. For example, azithromycin, acetaminophen, hydroxychloroquine, lopinavir, and remdesivir are considered to induce liver injury (Rodríguez-Morales et al., 2020; Sanders et al., 2020). Other drugs such as antibiotics, propofol, and corticosteroids also have hepatotoxic properties (Law et al., 2021; Sepehrinezhad et al., 2021). So far, the safety data on the drugs for treating COVID-19 patients with liver injury is still lacking, and all treatments are mainly based on past experience (Sun et al., 2020). Abnormal liver function is frequently observed in COVID-19 patients, and SARS-CoV-2 is closely related to liver injury or even liver failure (Li et al., 2020). Therefore, it is necessary to investigate whether liver injury in COVID-19 patients is associated with the direct effect of SARS-CoV-2, drugs used to treat COVID-19, underlying liver diseases, or more complicated disease courses. Growing epidemiological evidence has suggested that liver injury can occur in COVID-19 patients and may be associated with the therapeutic modalities used.

Drug‐induced liver injury (DILI) and other adverse events can lead to drug overdose in patients suffering from COVID-19 *via* impaired drug metabolism and elimination. Complications such as acute cardiovascular failure and kidney damage may also precipitate DILI in COVID-19 patients. In this study, we aimed to determine the incidence of liver injury in hospitalized patients with COVID-19 in China and explore whether these drugs are involved in DILI in these patients, so as to provide references for further research.

## Patients and Methods

### Study Design and Patients

This was a retrospective, multi-center case series study including patients from four hospitals–Tongji Hospital, Leishenshan Hospital, Zhongnan Hospital of Wuhan University, and Wuchang Hospital in Wuhan, China. Based on the WHO interim guideline (World Health Organization, 2020), 5,713 patients were diagnosed with COVID-19 from 19 December 2019 to April 26, 2020.

**FIGURE 1:**
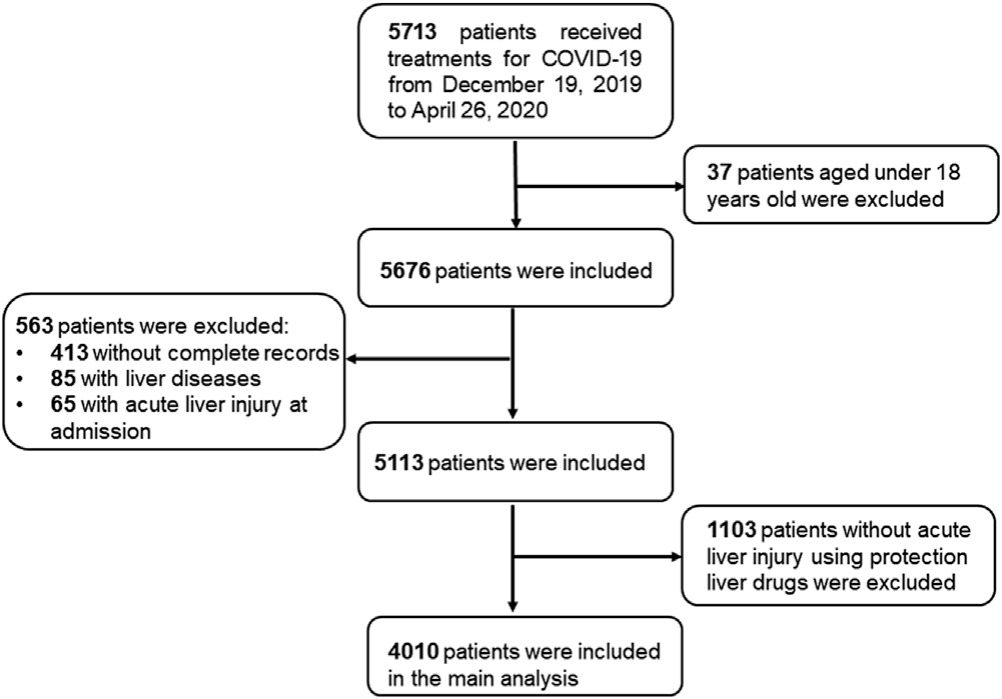
The flowchart showing the strategy of participant enrollment.

Among 5,713 patients, we excluded the following patients from the study: 1) 37 patients under the age of 18, 2) 563 patients with incomplete records in laboratory biomarkers or medications, or diagnosed with chronic liver diseases, or acute liver injury at admission, and 3) 1103 patients without liver injury who used protection liver drugs during hospitalization. The final analytic sample included 4010 patients receiving treatment from 19 December 2019 to April 26, 2020. (Figure 1).

### Acute Liver Injury Assessment

Acute liver injury was defined as alanine aminotransferase (ALT) or aspartate transaminase (AST) ≥ 3×upper Limit of Normal (ULN), alkaline phosphatase (ALP) or total bilirubin (TBIL) ≥ 2×ULN.

### Medications

In our analysis, medications used in the treatment of COVID-19 patients focused on oseltamivir, arbidol, interferon, ribavirin, lopinavir/ritonavir (LPV/r), hydroxychloroquine/chloroquine (HCQ/CQ), antibiotics, antifungals, corticosteroids, traditional Chinese medicine (TCM), parenteral nutrition (PN), enteral nutrition (EN), and immunotherapy.

### Covariates

According to the diagnosis and treatment plan for COVID-19 (Medicine, 2020), severity of COVID-19 was classified into 4 levels: mild, moderate, severe, and critically severe. Mild cases were defined as having few symptoms without CT findings. Moderate cases had fevers, respiratory symptoms and radiologic signs of pneumonia. Severe cases were defined by the presence of any of the following: 1) respiration rate ≥30 times/minute, 2) oxygen saturation SpO_2_ (resting state) ≤ 93%, 3) abnormal blood gas analysis: partial arterial oxygen pressure (PaO_2_)/fraction of inspired oxygen (FiO_2_) ≤ 300 mmHg. And critically severe cases were defined by the presence of any of the following: 1) respiratory failure that requires mechanical ventilation, 2) shock, 3) accompanied by other organ failure that needs ICU admission. These cases were further divided into two groups: non-severe group (mild cases and moderate cases) vs severe group (severe cases and critically severe cases).

In this study, the following covariates were included in the main analysis: age (years), sex (male or female), severity of COVID-19 at admission (severe or non-severe), initial symptoms (fever and cough, yes or no), diabetes (yes or no), hypertension (yes or no), cardiovascular disease (yes or no) and CVD (cerebrovascular disease) (yes or no).

### Statistical Analysis

Categorical variables were described by frequency (percentages), and continuous variables by mean (SD) or median (IQR). χ2 or Fisher’s exact test were used to compare the proportions in different subgroups; t tests or Mann-Whitney U test were used for continuous variables.

We used Cox proportional hazards regression with time-varying covariates to estimate the association of medications and acute liver injury. First, we examined the association by computing crude hazard ratio (HR). Second, we used an adjusted model that adjusted for age, sex, severity of COVID-19 at admission, fever, cough, hypertension, diabetes, cardiovascular disease, CVD and medications of interest. Note that all covariates about medications were time-varying. Furthermore, we compared the results of time-varying Cox models with those of logistic regression models in fully adjusted forms.

To assess whether the association between medications and DILI differed across admission severity groups, we examined the potential effect modification by admission severity (severe group vs non-severe group).

In addition, a sensitivity analysis with 1103 patients who did not have DILI but using liver protection drugs was performed to confirm the robustness of our results. The details can be seen in supplementary materials.

All analyses were performed in R version 4.0.2 software. And a two-tailed *p*-value <0.05 was considered statistically significant.

## Results

### Clinical Features of COVID-19 Patients at Admission

Among 4010 patients receiving treatment for COVID-19, 395 (9.85%) had acute liver injury during hospitalization. For all patients, the median age was 61 [IQR: (49, 69)] years old, the mean (SD) hospital stay was 21.49 (11.89) days, and the mean (SD) days for the first acute liver injury was 9.57 (9.38) days after admission.

Table 1 demonstrates the clinical features of COVID-19 patients at admission. Patients who developed DILI were predominantly male and had a longer hospital stay. Of them, 122 (5.64) were categorized in the non-severe group, while 273 (14.78) were in the severe group. The time from symptom onset to hospitalization was 9.57 (9.38) days. Common symptoms included fever (n = 238, 10.82%), followed by cough (n = 234, 10.34). The most common coexisting diseases included hypertension (n = 106, 9.22), diabetes (n = 95, 13.81), cardio-cerebrovascular diseases (n = 20, 8.44%), cardiovascular disease (n = 19, 19.59) and cancer (n = 9, 23.08). The occurrence rate of liver injury was slightly higher (*p* < 0.05) in males (n = 233, 12.6) than in females (n = 162, 7.5). COVID-19 patients with diabetes or cancer had higher liver injury-related morbidity than those without diabetes or cancer (*p <* 0.05). Cumulative DILI curve also showed that male or severe groups tended to develop DILI (Supplementary Figure S1).

**TABLE 1:** Characteristics of 4010 patients with COVID-19 at admission.

		Acute liver injury		
	Overall	No	Yes	*p* value
	N = 4010	N = 3,615	N = 395	
Age (median [IQR])	61.0 [49.0, 69.0]	61.00 [49.0, 69.0]	63.0 [49.0, 72.0]	0.023
Gender (%)				<0.001
Female	2161 (53.9)	1999 (92.5)	162 (7.5)	
Male	1849 (46.1)	1616 (87.4)	233 (12.6)	
Admission severity (%)				<0.001
Non-severe	2163 (53.9)	2041 (94.36)	122 (5.64)	
Severe	1847 (46.1)	1574 (85.22)	273 (14.78)	
Fever (%)				0.027
No	1810 (45.1)	1653 (91.33)	157 (8.67)	
Yes	2200 (54.9)	1962 (89.18)	238 (10.82)	
Cough (%)				0.258
No	1747 (43.6)	1586 (90.78)	161 (9.22)	
Yes	2263 (56.4)	2029 (89.66)	234 (10.34)	
Diabetes (%)				<0.001
No	3,322 (82.8)	3,022 (90.97)	300 (9.03)	
Yes	688 (17.2)	593 (86.19)	95 (13.81)	
Hypertension (%)				0.427
No	2860 (71.3)	2571 (89.9)	289 (10.1)	
Yes	1150 (28.7)	1044 (90.78)	106 (9.22)	
Cancer (%)				0.012
No	3,971 (99.0)	3,585 (90.28)	386 (9.72)	
Yes	39 (1.0)	30 (76.92)	9 (23.08)	
Cardio-cerebrovascular diseases (%)				0.494
No	3,773 (94.1)	3,398 (90.06)	375 (9.94)	
Yes	237 (5.9)	217(91.56)	20 (8.44)	
CVD (%)				0.002
No	3,913 (97.6)	3,537 (90.39)	376 (9.61)	
Yes	97 (2.4)	78 (80.41)	19 (19.59)	
Outcome (%)				<0.001
Death	190 (4.7)	89 (46.84)	101 (53.16)	
Recovery	3,820 (95.3)	3,526 (92.30)	294 (7.70)	
Hospital stay (mean (SD))	21.49 (11.89)	20.81 (11.01)	27.03 (15.17)	<0.001
Injury time (median [IQR])			9.57 (9.38)	

Abbreviations: CVD: cardiovascular disease. IQR, interquartile range.

### Medication for COVID-19 Patients During Hospitalization

Table 2 shows the use of medication during hospitalization. Note that for those who developed acute liver injury, the drugs were administered only before the first acute liver injury were considered.

**TABLE 2:** Medication for 4010 patients with COVID-19 during hospitalization.

Medicines	Acute liver injury		
	Overall	No	Yes	*p* value
	N = 4010	N = 3,615	N = 395	
Oseltamivir (%)				0.208
No	3,498 (87.2)	3,145 (89.91)	353 (10.09)	
Yes	512 (12.8)	470 (91.8)	42 (8.2)	
Arbidol (%)				0.127
No	1568 (39.1)	1399 (89.22)	169 (10.78)	
Yes	2442 (60.9)	2216 (90.75)	226 (9.25)	
Interferon (%)				0.973
No	3,540 (88.3)	3,192 (90.17)	348 (9.83)	
Yes	470 (11.7)	423 (90.0)	47 (10.0)	
Ribavirin (%)				<0.001
No	3,724 (92.9)	3,381 (90.79)	343 (9.21)	
Yes	286 (7.1)	234 (81.82)	52 (18.18)	
LPV/r (%)				0.041
No	3,614(90.1)	3,270 (90.48)	344 (9.52)	
Yes	396 (9.9)	345 (87.12)	51 (12.88)	
HCQ/CQ (%)				0.001
No	3,630 (90.5)	3,253 (89.61)	377 (10.39)	
Yes	380 (9.5)	362 (95.26)	18 (4.74)	
Antibiotic (%)				<0.001
No	1704 (42.5)	1602 (94.01)	102 (5.99)	
Yes	2306 (57.5)	2013 (87.29)	293 (12.71)	
Antifungal (%)				<0.001
No	3,940 (98.3)	3,570 (90.61)	370 (9.39)	
Yes	70 (1.7)	45 (64.29)	25 (35.71)	
Corticosteroids (%)				<0.001
No	3,006 (75.0)	2811 (93.51)	195 (6.49)	
Yes	1004 (25.0)	804 (80.08)	200 (19.92)	
TCM (%)				<0.001
No	524 (13.1)	381 (72.71)	143 (27.29)	
Yes	3,486 (86.9)	3,234 (92.77)	252 (7.23)	
PN (%)				<0.001
No	3,640 (90.8)	3,333 (91.57)	307 (8.43)	
Yes	370 (9.2)	282 (76.22)	88 (23.78)	
EN (%)				<0.001
No	3,598 (89.7)	3,283 (91.25)	315 (8.75)	
Yes	412 (10.3)	332 (80.58)	80 (19.42)	
Immunotherapy (%)				<0.001
No	3,193 (79.6)	2934 (91.89)	259 (8.11)	
Yes	817 (20.4)	681 (83.35)	136 (16.65)	

Abbreviations: LPV/r = Lopinavir/Ritonavir; PN, parenteral nutrition; EN, enteral nutrition; TCM, traditional Chinese medicine; HCQ/CQ, Hydroxychloroquine/chloroquine.

Drug treatments for hospitalized patients with COVID-19 mainly included antiviral drugs, antibacterial drugs, antifungal drugs, hydroxychloroquine/chloroquine, corticosteroids, traditional Chinese medicine (TCM), immunotherapy, and nutrition drugs.

In patients who developed acute liver injury, 293 (12.71) patients were treated with antibiotics and 25 (35.71) patients were treated with antifungal agents. As for antiviral drugs, 52 (18.18), 200 (19.92), 252 (7.23), 88 (23.78) and 80 (19.42) patients were treated with ribavirin, corticosteroids, TCM, parenteral nutrition (PN), and enteral nutrition (EN), respectively. A significant difference was observed between patients who used and those who did not use these drugs (*p* < 0.05). Patients who received hydroxychloroquine/chloroquine 362 (95.26) and TCM 3234 (92.77) were less likely to have acute liver injury.

### Association Between Acute Liver Injury and Medications by Multivariate Analysis

The risk factors of DILI in the population of subjects receiving COVID-19 treatment were analyzed through logistic regression models and time-varying Cox models (Table 3). Univariate analysis showed that the use of ribavirin, antibiotics, antifungal drugs, corticosteroids, PN, EN, TCM, and immunotherapy were significant factors that increased the incidence of liver injury. In the fully adjusted model: the HRs for acute liver injury in patients treated with antibiotics, antifungals, corticosteroids, EN and PN were 1.97 [95% CI: (1.55, 2.51)], 3.10 [95% CI: (1.93, 4.99)], 2.31 [95% CI: (1.80, 2.96)], 1.82 [95% CI: (1.31, 2.52)] and 2.71 [95% CI: (1.98, 3.71)], respectively.

**TABLE 3:** Association between hospitalized medication and risk of acute liver injury among 4010 patients with COVID-19.

Medicines	Crude HR(95%CI)	*p* value	Adjusted HR(95%CI)^a^	*p* value
**Oseltamivir**	1.24(0.81,1.92)	0.324	0.99(0.64,1.54)	0.956
**Arbidol**	1.00(0.80,1.26)	0.969	0.84(0.66, 1.06)	0.140
**Interferon**	0.91(0.57, 1.45)	0.696	0.62(0.39, 1.00)	0.052
**Ribavirin**	2.46(1.65, 3.66)	<0.001	1.38(0.91, 2.10)	0.116
**LPV/r**	1.76(1.17, 2.64)	0.007	1.07(0.71, 1.63)	0.765
**HCQ/CQ**	0.44(0.20,0.98)	0.046	0.48(0.21, 1.08)	0.077
**Antibiotic**	3.43(2.79, 4.22)	<0.001	1.97(1.55, 2.51)	<0.001
**Antifungal**	12.29(7.91,19.09)	<0.001	3.10(1.93, 4.99)	<0.001
**Corticosteroids**	4.24(3.42, 5.26)	<0.001	2.31(1.80, 2.96)	<0.001
**EN**	3.69(2.77, 4.91)	<0.001	1.82(1.31, 2.52)	<0.001
**PN**	6.29(4.79, 8.25)	<0.001	2.71(1.98, 3.71)	<0.001
**TCM**	0.69(0.56, 0.85)	<0.001	0.96(0.77, 1.18)	0.677
**Immunotherapy**	3.01(2.33, 3.88)	<0.001	1.21(0.91, 1.61)	0.168

Abbreviations: PN, parenteral nutrition; EN, enteral nutrition; TCM, traditional Chinese medicine; HCQ/CQ, Hydroxychloroquine/chloroquine; LPV/r = Lopinavir/Ritonavir; HR, hazard ratio; CI, confidence interval.

a Model was adjusted for age, gender, admission severity, fever, cough, diabetes, hypertension, cardiovascular disease, CVD, and medicines in the table above.

**FIGURE 2:**
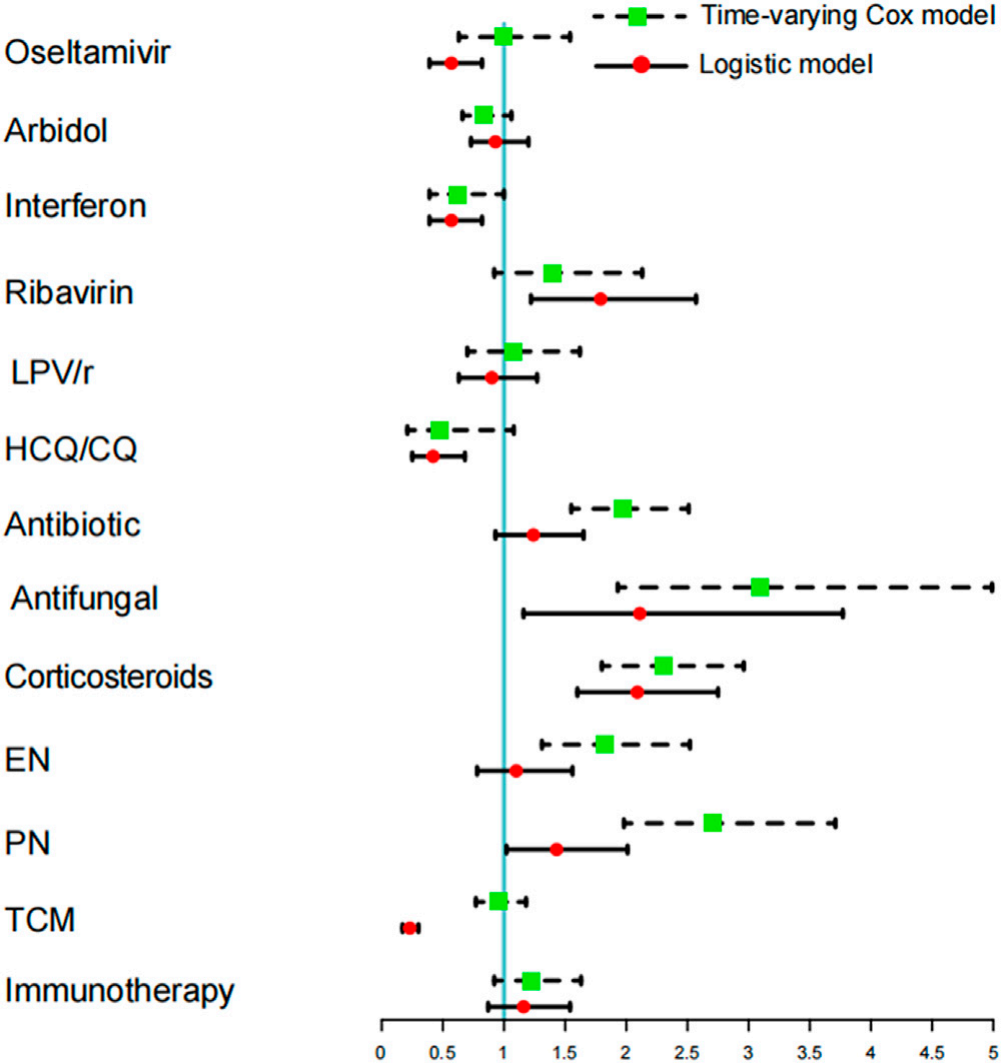
Comparing the time-varying Cox model and logistic regression model to study the different risks of acute liver injury between hospitalized medication and COVID-19 patients.

For comparison between time-varying Cox models and logistic regression models, both of them showed that antifungal, corticosteroids and PN could lead to a higher risk of DILI. Logistic regression models, however, underestimated the detrimental effects of antibiotics and EN while overestimating the protective effects of oseltamivir, interferon, HCQ/CQ, and TCM and the harmful effects of ribavirin (Figure 2).

The associations between hospital drugs and DILI in both severe and non-severe groups were determined (Supplementary Table S3). The detrimental effects of antibiotics, corticosteroids, and PN did not differ between severe and non-severe groups. In the sensitivity analysis including 1103 patients without DILI but using liver protection drugs, the results showed that antibiotics, antifungals, corticosteroids, EN and PN were risk factors for DILI, which were consistent with the results of the main analysis (Supplementary Table S4).

## Discussion

According to previous reports, the incidence of liver injury ranges from 14.8 to 53% in COVID-19 patients (Xu et al., 2020) and can reach up to 78% in severe COVID-19 patients (Zhang et al., 2020), which is mainly manifested by abnormal concentrations of aspartate aminotransferase and alanine aminotransferase. In this study, we found that the incidence of liver injury was 9.8%, slightly lower than that reported previously. This might be attributed to different types of patients admitted to the hospital and some patients who received liver protection drugs were excluded. In addition, the incidence of liver injury was remarkably higher in severe COVID-19 patients than in mild COVID-19 patients, which was consistent with previous reports (Cai et al., 2020; Guan et al., 2020). Besides, we observed that the risk of liver injury in patients suffering from COVID-19 was associated with the duration of hospital stay. The prolonged hospitalization could be due to the additional time needed for liver function recovery or the failure of virus eradication. Consistent with previous findings, men tended to have a high incidence of DILI. The reasons for this include gender differences in drug pharmacodynamics or pharmacokinetics; hormonal effects or interactions with signaling molecules or immunomodulators; and the immune system’s responses to certain drugs, reactive drug metabolites, or different adverse reactions of drug-protein adducts (Lucena et al., 2009; Björnsson et al., 2013). The incidence rates of liver injury in COVID-19 patients vary depending on their age, in which older patients have a higher incidence of liver injury. The impaired liver function in the elderly may increase the drug concentrations in their livers. Additionally, the decline in liver function can also explain the higher incidence of DILI in the elderly (Björnsson and Olsson, 2005; Bell and Chalasani, 2009).

In view of the unprecedented challenges brought by the pandemic, especially in the early stages when the admitted patients are seriously ill, effective therapeutic modalities are limited and the roles of possible co-infections remain unclarified; thus, antibiotics are widely used (Russell et al., 2021). However, we now know that bacterial co-infections are not common among community-acquired COVID-19 patients. Some studies demonstrated that most hospitalized patients with COVID-19 had secondary infections, which were acquired more than 2 days after hospital admission, and received one or more kinds of antimicrobials during their hospital stay. To our knowledge, antibiotics are the most common drug class that causes liver injury in the general population (Meier et al., 2005). Antibiotic-induced hepatotoxicity is often idiosyncratic and unpredictable, with unclear pathogenic mechanisms. In this study, antibiotics as an important risk factor for DILI in COVID-19 patients could be supported by previous literature reports, in which penicillin, sulfonamides, fluoroquinolones, tetracyclines, cephalosporins and macrolides were all related to liver injury (Bell et al., 2005; Andrade and Tulkens, 2011). In fact, the variation of DILI risk estimates depends greatly on the pharmacodynamic and pharmacokinetic properties of different antibiotics, which can affect the severity of liver injury (García Rodríguez et al., 1996; de Abajo et al., 2004; Andrade and Tulkens, 2011; Paterson et al., 2012).

Antifungal agent-induced liver injury has been proven to be a critical issue in COVID-19 patients with DILI. Most of the antifungal agents are associated with varying degrees of DILI. Humans and fungi are both eukaryotic organisms that share similar cellular enzymes and metabolic pathways. Such features are responsible for various adverse effects caused by numerous antifungal agents. Antifungal drugs, especially azoles, can affect liver metabolism. Cytochrome P450 enzyme complexes can be administered with other interacting drugs at the same time. This may lead to elevated serum levels of any interacting drugs, potential dose-related hepatotoxicity, and changes in the toxic metabolite profile. Severe liver injury with jaundice occurs most frequently after the administration of fluconazole, ketoconazole, terbinafine or voriconazole (Elewski and Tavakkol, 2005; Song and Deresinski, 2005; Perveze et al., 2007).

COVID-19 is shown to be associated with diffuse alveolar damage in the lung. Corticosteroids can mediate inflammation-related lung injury and reduce the progression from respiratory distress to death. Many guidelines for COVID-19 treatment have noted that corticosteroids may be contraindicated and are not recommended, even though in China, corticosteroids are only used to treat severe COVID-19 patients (Dagens et al., 2020). However, clinical practices vary considerably across the globe; in some countries, up to 50% of patients were administered with corticosteroids. Previous studies showed that patients treated with large amounts of hormones and combinations of multiple drugs (*p* = 0.031 and *p* = 0.002, respectively) were more susceptible to liver injury (Yao et al., 2020). This can be the reasons why liver injury occurs frequently in severe COVID-19 patients, since fewer drugs are administered to mild COVID-19 patients. It has been reported that the early stages of COVID-19 are characterized by the decreased levels of CD3^+^/CD4^+^/CD8^+^ cells (12). Hence, the administration of high-dose corticosteroids can worsen COVID-19 in immunosuppressed patients, which increases the risk of severe secondary infections (Wang et al., 2020). As a result, the use of antibiotics was increased. In addition, there is a possibility of drug-drug interactions. Some corticosteroids, such as dexamethasone, are moderate inducer of cytochrome P450 (CYP) 3A4, which can affect the concentrations and effects of other drugs or CYP3A4 substrates. Previous studies found that drugs metabolized by CYP enzymes (e.g., CYP1A2/2C8/2C9/3A5) had a 4-fold higher risk of DILI than those poorly metabolized by CYP enzymes (Andrade et al., 2009).

Hospital malnutrition and nutritional risk are common in patients with COVID-19 due to various factors such as inflammation-related hypermetabolism, mechanical ventilation, low dietary intake, gastrointestinal intolerance and lack of nutrition support service. COVID-19 patients in the ICU often require invasive or non-invasive ventilation and have other contraindications to enteral feeding that can affect treatment outcomes (Barazzoni et al., 2020; Yu et al., 2021). Nutritional support, including PN and EN, is recommended for critically ill patients to avoid the detrimental consequences of malnutrition. This indicated that patients treated with nutritional support have a risk of underfeeding and becoming severely ill (Kumar and Teckman, 2015). It has been reported that compared with critically ill patients, the ratio of critically ill patients receiving EN + PN or PN alone is higher (Barazzoni et al., 2020). During PN treatment, the luminal content was reduced, and the loss of hepatoprotective gut-derived signals contributed to PN-associated cholestasis (PNAC) (Madnawat et al., 2020). PNAC is commonly known as PN-associated liver disease or intestinal failure-associated liver disease (Lacaille et al., 2015). Therefore, nutraceuticals are the risk factors for liver damage, which is consistent with our findings.

Although it has been reported that some antiviral drugs such as ribavirin, arbidol, lopinavir and TCM can cause liver damage (Lopinavir, 2012; Chalasani et al., 2014), no risk factors for liver damage were found in our study. The reasons may be due to the difference in inclusion and exclusion standards and statistical methods.

Cox proportional hazards regression with time-varying covariates can take daily medication use into consideration; thus, it is better than conventional logistic regression models in estimating the association between DILI and medications use. Although the coefficients are not always the same, both of them show that antifungals, corticosteroids, and PN can increase the risk for DILI. Given the changes in the daily use of antibiotics and EN, Cox regression analysis reveals that they are significant risk factors of DILI. In addition, considering the uncertainty around the impact of liver protection drugs on liver function recovery, we included 1103 patients who did not receive the above-mentioned drugs, but instead used liver protection drugs, in a sensitivity analysis to prove the reliability of our findings. The obtained results are still consistent with our main analysis and have good robustness.

## Limitations

There are several limitations to this study. First, a retrospective study may suffer from selection bias. Although we adjusted for many potential confounders, we cannot exclude the possibility of residual confounders. Second, because the patients in this study were only recruited from four hospitals in Hubei province, our results may not be generalizable to other regions. Third, our study did not consider the impacts of drug dosage, drug-drug interaction, and changes in disease severity during hospitalization. Finally, the effects of different treatment regimens on the severity of liver damage were not determined.

## Conclusion

The incidence of DILI was considerably high in hospitalized patients with COVID-19. The uses of antibiotic, antifungal, corticosteroids, EN and PN were significant risk factors for liver injury in COVID-19 patients. The risk of adverse reactions can be minimized by selecting appropriate medications and paying attention to indications and contraindications in each case. Health professionals should closely monitor these risk factors during hospitalization, adjust the drug’s dosage, and limit the medications used in time.

## Data Availability

The original contributions presented in the study are included in the article/Supplementary Material, further inquiries can be directed to the corresponding authors.
