# Supplementary material for: Association Between Drug Treatments and the Incidence of Liver Injury in Hospitalized Patients With COVID-19
Source: Front Pharmacol. 2022 Mar 21;13:799338. doi: 10.3389/fphar.2022.799338 (PMC8978013; doi:10.3389/fphar.2022.799338)
Supplement: Supplementary file 1 [file Table1.docx]

**Table S1.** Liver test results of 4010 patients with COVID-19 during hospitalization.

|  |  | **Acute liver injury** | |  |
| --- | --- | --- | --- | --- |
|  | **Overall** | **No** | **Yes** | **P value** |
|  | **N=4010** | **N=3615** | **N=395** |  |
| ALP (Median [IQR]) | 75.0 [62.0;94.0] | 73.0 [61.0;89.0] | 111 [81.0;184] | <0.001 |
| ALT (Median [IQR]) | 27.0 [18.0;43.0] | 25.0 [17.0;38.0] | 152 [90.4;218] | <0.001 |
| AST (Median [IQR]) | 24.0 [18.0;35.0] | 23.0 [18.0;31.0] | 101 [62.5;159] | <0.001 |
| GGT (Median [IQR]) | 31.0 [20.0;54.0] | 29.0 [19.0;47.0] | 112 [56.5;190] | <0.001 |
| TBIL (Median [IQR]) | 10.8 [8.30;14.5] | 10.5 [8.10;13.9] | 16.4 [11.1;27.2] | <0.001 |

Abbreviations: Abbreviations: ALT = alanine aminotransferase; AST = aspartate aminotransferase; GGT = glutamyl transferase; ALP = alkaline phosphatase; TBIL = total bilirubin
