# Supplementary material for: Association Between Drug Treatments and the Incidence of Liver Injury in Hospitalized Patients With COVID-19
Source: Front Pharmacol. 2022 Mar 21;13:799338. doi: 10.3389/fphar.2022.799338 (PMC8978013; doi:10.3389/fphar.2022.799338)
Supplement: Supplementary file 2 [file Table2.docx]

**Table S2.** Clinical classification and prognosis of 395 patients with COVID-19 after acute liver injury.

| **Characteristic** | **Acute liver injury**  **Total = 395** |
| --- | --- |
| **Clinical pattern of liver injury**^†^ |  |
| Hepatocellular | 112 |
| Cholestatic | 122 |
| Mixed | 159 |
| **Recovery after different degrees of liver injury*** |  |
| Mild | 151 |
| Moderate | 105 |
| severe | 71 |

† Hepatocellular: R≥5; Cholestasis: R≤2; Mixed: 2<R<5;

R = (Measured value of ALT/ALT ULN)/(Measured value of ALP/ALP ULN)

* Mild: liver function indicators returned to normal; Moderate: liver function indicators decreased by >50% compared to before treatment; severe: liver function indicators did not improve or worsen.
