# Supplementary material for: Association Between Drug Treatments and the Incidence of Liver Injury in Hospitalized Patients With COVID-19
Source: Front Pharmacol. 2022 Mar 21;13:799338. doi: 10.3389/fphar.2022.799338 (PMC8978013; doi:10.3389/fphar.2022.799338)
Supplement: Supplementary file 3 [file Table3.docx]

**Table S3.** Associations between hospital drugs and risk of acute liver injury in different admission severity (severe group vs. non-severe group) among 4010 patients with COVID-19.

|  | **Non-severe group** | | **Severe group** | |
| --- | --- | --- | --- | --- |
|  | **Adjusted HR(95%CI) ^†^** | **P value** | **Adjusted HR(95%CI)^†^** | **P value** |
| **Oseltamivir** | 1.55(0.86, 2.77) | 0.142 | 0.49(0.22, 1.11) | 0.087 |
| **Abidor** | 0.62(0.38, 1.01) | 0.054 | 0.93(0.70, 1.22) | 0.581 |
| **Interferon** | 0.96(0.47, 1.97) | 0.920 | 0.50(0.26, 0.97) | 0.040 |
| **Ribavirin** | 1.85(0.88, 3.90) | 0.105 | 1.24(0.74, 2.08) | 0.414 |
| **LPV/r** | 0.81(0.19, 3.40) | 0.775 | 1.05(0.68, 1.63) | 0.832 |
| **HCQ/CQ** | 0.34(0.05, 2.42) | 0.278 | 0.55(0.23, 1.36) | 0.196 |
| **Antibiotic** | 2.10(1.39, 3.16) | <0.001 | 1.93(1.43, 2.61) | <0.001 |
| **Antifungal** | 3.04(0.41, 22.46) | 0.276 | 3.13(1.88, 5.21) | <0.001 |
| **Corticosteroids** | 2.54(1.54, 4.19) | <0.001 | 2.13(1.59, 2.84) | <0.001 |
| **EN** | 2.01(0.60, 6.69) | 0.256 | 1.76(1.24, 2.49) | 0.001 |
| **PN** | 3.02(1.41, 6.47) | 0.004 | 2.56(1.80, 3.64) | <0.001 |
| **TCM** | 1.44(0.96, 2.17) | 0.076 | 0.79(0.61, 1.02) | 0.067 |
| **Immunotherapy** | 0.85(0.38, 1.90) | 0.694 | 1.30(0.95, 1.78) | 0.095 |

Abbreviations: PN = parenteral nutrition; EN = enteral nutrition; TCM = traditional Chinese medicine; HCQ/CQ = Hydroxychloroquine/chloroquine; LPV/r = Lopinavir/Ritonavir; HR = hazard ratio; CI = confidence interval.

† Model was adjusted for age, gender, fever, cough, diabetes, hypertension, cardiovascular disease, CVD and medicines in the table above.
