# Supplementary material for: Association Between Drug Treatments and the Incidence of Liver Injury in Hospitalized Patients With COVID-19
Source: Front Pharmacol. 2022 Mar 21;13:799338. doi: 10.3389/fphar.2022.799338 (PMC8978013; doi:10.3389/fphar.2022.799338)
Supplement: Supplementary file 4 [file Table4.docx]

**Table S4.** Associations between hospital drugs and risk of acute liver injury among 5113 patients with COVID-19.

|  | **Crude HR(95%CI)** | **P value** | **Adjusted HR(95%CI)^†^** | **P value** |
| --- | --- | --- | --- | --- |
| **Oseltamivir** | 1.06(0.89,1.63) | 0.802 | 0.88(0.57,1.38) | 0.588 |
| **Abidor** | 0.98(0.79, 1.23) | 0.876 | 0.87(0.69, 1.10) | 0.248 |
| **Interferon** | 0.87(0.55, 1.38) | 0.552 | 0.62(0.39, 1.00) | 0.050 |
| **Ribavirin** | 2.26(1.52, 3.37) | <0.001 | 1.44(0.95, 2.19) | 0.088 |
| **LPV/r** | 1.38(0.92, 2.07) | 0.125 | 0.88(0.58, 1.34) | 0.557 |
| **HCQ/CQ** | 0.46(0.21, 1.04) | 0.062 | 0.45(0.20, 1.01) | 0.053 |
| **Antibiotic** | 3.03(2.47, 3.73) | <0.001 | 1.83(1.44, 2.32) | <0.001 |
| **Antifungal** | 10.41(6.70,16.17) | <0.001 | 3.02(1.88, 4.85) | <0.001 |
| **Corticosteroids** | 3.60(2.91, 4.47) | <0.001 | 2.21(1.73, 2.81) | <0.001 |
| **EN** | 3.46(2.60, 4.59) | <0.001 | 1.62(1.17, 2.26) | 0.004 |
| **PN** | 5.75(4.38, 7.54) | <0.001 | 2.75(2.02, 3.75) | <0.001 |
| **TCM** | 0.70(0.57, 0.86) | 0.001 | 0.91(0.74, 1.13) | 0.393 |
| **Immunotherapy** | 2.72(2.11, 3.51) | <0.001 | 1.26(0.95, 1.67) | 0.112 |

Abbreviations: PN = parenteral nutrition; EN = enteral nutrition; TCM = traditional Chinese medicine; HCQ/CQ = Hydroxychloroquine/chloroquine; LPV/r = Lopinavir/Ritonavir; HR = hazard ratio; CI = confidence interval.

† Model was adjusted for age, gender, admission severity, fever, cough, diabetes, hypertension, cardiovascular disease, CVD and medicines in the table above.
